# Microsurgical resection of a scalp arteriovenous fistula with intracranial drainage: an illustrative case

**DOI:** 10.3171/2025.7.FOCVID25109

**Published:** 2025-10-01

**Authors:** Abdullah Keles, Ufuk Erginoglu, Mete Ugur, Kelsey Bowman, Beverly Aagaard-Kienitz, Mustafa K. Baskaya

**Affiliations:** Department of Neurological Surgery, University of Wisconsin School of Medicine and Public Health, Madison, Wisconsin

**Keywords:** cirsoid aneurysm, emissary vein, occipital artery, open vascular surgery, scalp arteriovenous fistula, superior sagittal sinus

## Abstract

Scalp arteriovenous fistulas are abnormal connections between scalp arteries and draining veins without an intervening capillary bed. They may present as discolored nodules or pulsatile soft scalp lesions. These lesions are most commonly fed by the superficial temporal and occipital arteries with venous drainage typically occurring through extracranial veins. Intracranial venous drainage is rare. Management options include surgical excision, endovascular embolization, or direct intralesional sclerosing agent injection. Here the authors present a case of a scalp arteriovenous fistula with intracranial drainage treated with microsurgical resection by an expert interdisciplinary team, highlighting the importance of collaborative, tailored treatment strategies for optimal outcomes.

The video can be found here: https://stream.cadmore.media/r10.3171/2025.7.FOCVID25109

## Transcript

This video demonstrates an illustrative case of a microsurgical resection of a scalp arteriovenous fistula with intracranial drainage.

### 0:27 Introduction.

Scalp arteriovenous fistulas are rare vascular anomalies characterized by direct connections between the scalp arteries and veins, bypassing the capillary bed. They typically present as discolored or pulsatile scalp lesions and are most often supplied by the superficial temporal or occipital arteries. While venous drainage is usually extracranial, intracranial drainage is uncommon and requires special consideration.

### 0:49 Case Presentation.

A 26-year-old male patient presented with a red bump on his scalp that he first noticed 2 years earlier, accompanied by progressively worsening headaches. The patient was evaluated in our interdisciplinary neuroendovascular surgery outpatient clinic by both endovascular and open surgical specialists. On admission, the patient was neurologically intact. Physical examination revealed a palpable, erythematous lump on the left posterior occiput, measuring up to 5 cm in maximum dimension. He had no history of trauma to the area and no significant past medical, surgical, or family history.

### 1:23 Preoperative Imaging.

Initial magnetic resonance imaging showed a tangle of vessels over the left parieto-occipital calvarium, most consistent with a scalp arteriovenous fistula. Selective digital subtraction angiography was performed with injections of the internal carotid, external carotid, occipital, and vertebral arteries. The multiple injections demonstrated a dural arteriovenous fistula with two prominent fistulous points from branches off of the occipital artery. No additional feeders were identified. The fistula drains into an emissary vein that ultimately transosseously draining into the superior sagittal sinus.

### 1:57 Management.

The patient was evaluated in the neuroendovascular surgery clinic by both endovascular and open microsurgical experts to ensure the best possible outcome. Embolization alone was deemed high-risk due to intracranial drainage into the superior sagittal sinus. Open surgical resection would allow for resection of the involved pathologic vessels and definitive clipping of the emissary vein draining the fistula. The decision to proceed with the goal of complete resection of the arteriovenous fistula; if not possible, disconnect the emissary vein connection. Depending on the completeness of the surgical resection, postoperative embolization would be considered. After discussing the findings and treatment options with the patient and his family, the patient elected to proceed with surgery.

### 2:39 Patient Positioning.

The patient was positioned supine and anesthesia was induced. His head was then secured in a head clamp and turned to the right. The hair overlying the incision site was clipped.

### 2:48 Intraoperative Doppler Ultrasound.

Intraoperative Doppler ultrasound was used to map the vascular malformation on the scalp. The incision and trajectory of the occipital artery and lesion were then marked accordingly. Radiopaque markers were placed along the incision to assist with angiographic guidance.

### 3:01 Intraoperative Intravenous Digital Subtraction Angiography.

Then intraoperative intravenous digital subtraction angiography was performed to confirm the most accurate location of the emissary vein. The needle laser localization feature of the angiography machine was used to pinpoint the exact location of the emissary vein fistulous connection, which was marked on the skin. An incision was planned along the course of the occipital artery and wrapping around the main tortuous vessels, continuing a few centimeters beyond the draining emissary vein. In this particular case, the incision was made around the erythematous bulb to minimize intraoperative bleeding. The head was then carefully prepped and draped in the usual sterile fashion. The remainder of the procedure was performed using standard microsurgical techniques.

### 3:42 Skin Incision.

The skin was sharply opened with a 15 blade beyond the expected location of the draining vein. We carefully dissected down through the subcutaneous tissue and galea using the electrocautery and microscissors.

After careful dissection, we were able to identify a small opening in the bone slightly medial to our incision. Then intraoperative Doppler ultrasound was used to confirm the most accurate location of the emissary vein. This was carefully exposed, and large venous structure draining into this bony defect was noted.

### 4:10 Occipital Artery Dissection.

We then turned our attention to the proximal portion of the incision along the expected course of the occipital artery. Then intraoperative Doppler ultrasound was used to confirm the most accurate location of the occipital artery. Dissection proceeded in a similar fashion until the enlarged and tortuous occipital artery was encountered. We followed the occipital artery distally along its course into the area where it entered the region of erythematous thickened scalp. We were unable to dissect the occipital artery and associated malformation from the overlying skin, so the decision was made to excise the entire region, including the full-thickness scalp.

Prior to completely removing this abnormal vascular malformation within the scalp, a temporary clip was placed in the occipital artery and then the occipital artery was bipolared and divided. Then the abnormal-appearing scalp, 3 by 4 cm in size, was ellipsed out and removed en bloc.

### 5:08 Draining Vein Coagulation.

Once the entirety of the vascular malformation had been removed, attention was returned to the draining emissary vein. This was thoroughly coagulated and clipped with two Weck clips, which were also serve as markers for potential future intervention and imaging. At this stage, resection of the scalp vascular malformation and disconnection of the fistulous point were complete, and we proceeded with closure.

### 5:23 Closure.

Skin closure was particularly complex and difficult due to the extent of scalp resection required to remove the portion of the vascular malformation involving the overlying skin. Therefore, radial scoring of the galea was performed to allow for additional scalp mobilization.

After mobilizing the scalp, we were able to reapproximate the incision edges. Remainder of the incision was closed in standard fashion.

### 5:47 Early Postoperative Course.

The patient awoke without any neurological deficits. His hospital stay was uneventful, and he was discharged home on postoperative day 1. Histopathological analysis confirmed the diagnosis.

### 5:56 Outpatient Follow-Up.

The patient was followed postoperatively by both endovascular and open vascular teams. By the 3-month follow-up, the patient’s headaches had completely resolved. At the 2-year follow-up, postoperative magnetic resonance imaging and digital subtraction angiography confirmed complete resection of the arteriovenous fistula with no evidence of recurrence.

### 6:15 Discussion.

Scalp arteriovenous fistulas are rare vascular lesions that may be congenital, acquired, or iatrogenic. Their management requires careful evaluation and individual treatment planning by an expert interdisciplinary team. They may present as discolored nodules to as an enlarging, pulsatile, soft compressible lesions on the scalp. Associated symptoms include local pain, headaches, and tinnitus.

On physical examination, palpable thrill and audible bruit are commonly present, and scalp discoloration may also be observed. External compression of feeding arteries may reduce the size, pulsation, and thrill of the lesion. If left untreated, they may lead to hemorrhage and scalp necrosis. They are most commonly fed by the superficial temporal and occipital arteries, but may also receive contributions from the ophthalmic, facial, and maxillary arteries.^1^

Venous drainage typically occurs through extracranial veins such as superficial temporal, preauricular, facial, angular, ophthalmic, and external jugular veins.^1,2^ Intracranial venous drainage is rare and requires special consideration in management.

Computed tomography and magnetic resonance angiography provide valuable information, but selective digital substruction angiography remains the gold standard for diagnosis. Depending on location, selective angiography of potential feeding vessels should be obtained.^3^ Differential diagnosis includes dural arteriovenous fistulas, sinus pericranii, hemangiomas, hamartomas, and neoplasms.

Treatment is indicated for cosmetic concerns, prevention of hemorrhage and scalp necrosis, and symptom relief. Management options include surgical excision, endovascular embolization, or direct intralesional injection of sclerosing agents. The choice of treatment typically depends on the unique features of the lesion and the expertise of the treatment providers.

Historically, surgical excision was standard of care. Complete excision of the draining vein is essential, as incomplete removal has been associated with frequent recurrence.^4,5^ Therefore, accurate identification and localization of all draining veins is of utmost importance, as seen in our case. Inaccurate preoperative localization of feeding vessels may result in excessive intraoperative blood loss.

Intraoperative Doppler ultrasound can help in planning the optimal scalp incision, as we demonstrated in our case. With advancements in less invasive endovascular techniques, more treatment options have become available. These include transarterial or transvenous embolization, which are now frequently employed.

Large draining veins and high-flow fistulas require special attention, as embolization in such cases may result in migration of embolizing agent into venous sinuses or pulmonary vessels, leading to life-threatening complications.^1,6,7^

Endovascular treatments may also carry a risk of scalp necrosis.^1^ Small lesions, lesions with few feeding and draining vessels can be ideal candidates for endovascular treatments.^5^ Definitive endovascular treatment requires elimination of the draining veins.^4^ However, embolization of small feeding arteries can be technically challenging and may lead to recurrence if not eliminated.^8^

In combined treatment approaches, preoperative embolization can facilitate surgery by reducing blood loss. Direct intralesional injection of sclerosing agents may also provide alternative treatment in selected cases.^9^

Given the variety of available treatment modalities, management should involve a multidisciplinary expert team of both endovascular and open surgical specialists to ensure the best possible outcome for each patient.

### 9:37 Conclusions.

In conclusion, scalp arteriovenous fistulas are rare vascular lesions requiring individualized management based on feeding artery and draining venous patterns. In our case, microsurgical resection of a scalp arteriovenous fistula with intracranial drainage into the superior sagittal sinus was presented. Intraoperative Doppler ultrasound and intravenous digital subtraction angiography were essential for accurate localization. Complete resection, including disconnection of the emissary vein, eliminated the need for postoperative embolization. This case highlights the importance of thorough preoperative planning and interdisciplinary collaboration to ensure safe and effective treatment in complex arteriovenous fistulas.

## Acknowledgments

We thank Steven L. Goodman, PhD, distinguished editor, for his editorial assistance.

## Disclosures

The authors report no conflict of interest concerning the materials or methods used in this study or the findings specified in this publication.

## Author Contributions

Primary surgeon: Baskaya. Assistant surgeon: Bowman. Editing and drafting the video and abstract: Keles, Erginoglu. Critically revising the work: Baskaya, Keles, Erginoglu, Bowman. Reviewed submitted version of the work: Baskaya, Keles, Ugur, Bowman. Approved the final version of the work on behalf of all authors: Baskaya. Supervision: Baskaya. Neuroendovascular surgeon: Aagaard-Kienitz.

## Supplemental Information

### Patient Informed Consent

The necessary patient informed consent was obtained in this study.
